# Exploring the perceptions of how living with osteoarthritis affects employed people's work productivity

**DOI:** 10.1002/msc.1739

**Published:** 2023-02-04

**Authors:** Angela Ching, Yeliz Prior

**Affiliations:** ^1^ School of Health and Society Centre for Human Movement and Rehabilitation University of Salford Salford Greater Manchester UK

**Keywords:** absenteeism, employment, osteoarthritis, presenteeism, qualitative, work accommodations, workplace support

## Abstract

**Background:**

Working people with osteoarthritis (OA) can experience difficulties at work due to pain and activity limitation.

**Objective:**

To explore the impact of biopsychosocial factors on work participation in employed people with OA.

**Methods:**

An explorative, qualitative, semi‐structured one‐to‐one telephone interview study. Employed people living with OA were recruited through an arthritis volunteer database and social media. Data was analysed using Thematic Analysis.

**Findings:**

Participants (*n* = 15) included people with OA in any joints from mixture of careers. Themes were: (1) Effects of OA on work participation, (2) Impact of workplace support and accommodations on employment, and (3) Importance of clinical support and management.

**Conclusion:**

Impact of OA on people's work productivity results in a spill over into their home lives. Work transitions and adaptations are not always available. Employers need to be educated to support employees to create a positive work environment and promote available workplace support.

## 1 INTRODUCTION

Osteoarthritis (OA) is the commonest form of arthritis. OA can occur on single or multiple joints, but most commonly affects the knee, hip or finger joints (Arthritis Research UK, 2013). It causes the joints to become painful, swell and can limit their movement (Versus Arthritis, 2021). People of the working age living with OA can experience difficulties carrying out their work tasks, such as travelling to and from work, prolonged periods of sitting or standing, or using a keyboard due limitation caused by pain and mobility (Versus Arthritis, 2021). A recent online survey of people with OA (*n* = 1041) found 61% of those still working reported they might have to retire early due to OA; 19% of respondents said that they had already reduced their working hours because of their OA; and 17% had already retired early (Versus Arthritis, 2021).

Furthermore, recent studies have highlighted the importance of exploring the biopsychosocial and work‐related factors on productive employment in people living with musculoskeletal conditions. Agaliotis et al., examined whether demographic, psychological or workplace factors influence if older people with chronic knee pain remain in productive employment (Agaliotis et al., 2018). The common challenges for continuing employment were the extent of physical limitations due to chronic knee pain, types of work transitions utilised to accommodate knee pain, lack of ergonomic workplace policies, complexity of disclosure, social support at work and the unpredictability of future arthritis progression (Agaliotis et al., 2018). However, this study did not explore the effects of work spillover (i.e., the stresses experienced in one area of life (e.g., taking care of family, work/life balance or financial concerns) leading to stresses in another area of life (e.g., managing OA), and vice versa (Agaliotis et al., 2018; Gignac et al., 2006)). Oakman et al., explored work and non‐work supports used by employees who live with persistent musculoskeletal pain to assist them in maintaining productive employment (Oakman et al., 2017). Organisational factors, physical work environment and personal limitations were barriers to working productively (Oakman et al., 2017). The key determinants of maintaining sustainable and productive employment were having a supportive employer and flexibility in the work organisation, but disclosure to an employer was a significant barrier to employment (Oakman et al., 2017). People with chronic knee pain continue to work without seeking adaptations or disclosing their condition to colleagues and supervisors (Kaptein et al., 2013). There is scope to explore why people choose to seek adaptations or disclose their condition with their employer and or colleagues.

The number of qualitative studies exploring the effects of OA on work ability, productivity, the use of work accommodations and choices to disclose their condition at work are scarce. A recent systematic review of qualitative studies exploring the experience of living with knee OA found 13 of 21 studies recorded details on participants' employment status (Wallis et al., 2019); but most participants were not working or retired, except for only in three studies (Darlow et al., 2018; Keysor et al., 1998; MacKay et al., 2016). Despite these studies recruiting participants who were employed, two of the studies did not explore the impact of OA on productive work (Darlow et al., 2018; MacKay et al., 2016); and only one of the three studies briefly explored the impact of OA on athletic young and middle‐aged adults' work (Keysor et al., 1998). One participant described they were unable to work 8 h a day as a physical therapist, and another participant needed to sit down for their work tasks as they were unable to stand for short periods of time (Keysor et al., 1998). With an ageing population, increasing retirement age, and an increase in prevalence of people affected by OA in the workforce, the aim of our study is to explore the impact of biopsychosocial and work‐related factors on work participation in employed people living with OA and access to workplace adaptations to remain in paid employment.

## 2 METHODS

### 2.1 Study design

Due to explorative nature of the research question, we have conducted a qualitative study with semi‐structured one‐to‐one telephone interviews. Critical realist theory was used as a framework to interpret the findings, which acknowledges that what was said by a participant is dependent on their own context, and how individuals experience reality depends on various factors, such as sociodemographic, biological, culture, language and interests (Clarke & Braun, 2013).

### 2.2 Ethical approval and participant consent

The University of Salford Health Sciences Research Ethics Panel approved the study (HSR1819‐008) and written informed consent was received from all participants.

### 2.3 Participant selection and eligibility criteria

Convenience sampling was used as it was low‐cost and easy, with potential participants being readily available. Participants were recruited via two routes: (1) Past study participants: people with OA who completed the work‐patient reported outcome measures (Work‐PROMs) study (Hammond et al., 2022) and agreed to be contacted for future research were invited to take part in this interview study and (2) Social Media: recruitment poster with study information was tweeted from the study's Twitter account.

Potential participants were provided with an invitation letter, a participant information sheet, sample consent form and an expression of interest (EOI) form via post or email. The EOI form asked them to complete the questions about themselves (to check their eligibility) and state if they were interested in taking part in the study. On receipt of the EOI form, the first author contacted the participant by telephone to explain the purpose of the study, confirm eligibility and answer any questions the potential participant may have before going through the informed consent process. Participants were posted or emailed the actual consent form to read, complete, sign and post or email back to the first author. Participants who do not meet the eligibility criteria were informed of the reasons and kindly thanked for their interest and time.

To be eligible, participants needed to be >18 years old; currently self‐employed or in paid employment for at least 1 day a week (they may also currently be on sick leave due to OA); employed for at least 6 months in their current job to ensure that the individual has had enough time to settle into their role and for any associated work problems to become apparent; self‐reported clinician‐diagnosis of OA, with OA being the primary condition that the participant perceives as having the main impact on their work participation; able to read, write, understand and speak in English to provide informed consent and take part in the interview.

### 2.4 Data collection

A semi‐structured topic guide was developed by the study team (Table 1) and piloted on three participants with no changes made. Interview questions were based around the aspects of their jobs they find difficult to perform, their use of sick leave, coping strategies, workplace adaptations and workplace support. All participants were interviewed by the first author (Angela Ching; experienced in qualitative research) by telephone. Participants did not know the interviewer prior to approach about the study. Field notes were taken during and after the interviews which helped the to reflect on the participants' experiences during data analysis.

**TABLE 1:** Semi‐structured interview schedule.

1. Can you tell me what joint(s) you have osteoarthritis (OA) and how long you have had OA for?
2. Can you describe your job title, nature of your job and typical workday?
3. What aspects of your work tasks do you find difficult to perform due to your arthritis?
a. Are there any other barriers that stops you from carrying out your work tasks? What are they and how do they impact your work participation?
4. Have you taken sick leave due to your OA? If yes, can you tell me a bit more about this?
5. Has there been times where you have carried on working but felt you struggled to meet your work demands due to your OA? If yes, what were the reasons behind continuing to go to work and what aspects of work were you struggling with?
6. Have you made any workplace adaptations/adapted work tasks to make working easier? If so, can you describe what these adaptations are and how helpful they have been?
7. Do you have any coping strategies you use at work and at home to manage your OA and work?
a. Can you describe your coping strategies?
8. What kind of support (if any) are you receiving from your line manager and colleagues? How do you feel about this?
a. Are you aware of any workplace policies that can give you support?
b. If you are not receiving any support, what kind of support would you like from your employers?
9. What kind of support (if any) are you receiving from health professionals? How do you feel about this?
10. Do they anticipate leaving their job early/changing jobs/changing their hours because of their OA? Why?

Preliminary data analysis was completed after each interview before a new participant is invited for their interview to ensure that when data saturation is reached (i.e., no new themes arise from the current data), no further interviews are conducted. The interviews were audio‐recorded and transcribed verbatim with names replaced by pseudonyms. Transcripts were checked for accuracy against the audio‐recordings.

### 2.5 Data analysis

All transcripts were independently coded by Angela Ching and Yeliz Prior using thematic analysis by Braun and Clarke (2019) including: (1) reading and re‐reading the transcripts to familiarise self with data, and initial codes/noting potential themes arising from the data; (2) re‐reading, and systematically coding the data; as each new code is identified, earlier transcripts were reviewed for these new codes (constant comparison); codes were organised into groups as themes were identified; (3) themes were defined in more detail and inter‐relationships established—steps 1–3 were an iterative process; and (4) themes were organised into a report, giving the meaning of the participants' experiences, grounded in their own words. An inductive approach to data analysis was used. Angela Ching and Yeliz Prior came together to discuss, develop and agree on the final themes and sub‐themes. Data were managed using NVivo 12 software (QSR International Pty Ltd.).

### 2.6 Research team and reflexivity

The research team were all female, academics and/or occupational therapist (Yeliz Prior), with interest in rheumatology and musculoskeletal conditions. All researchers (Angela Ching, Yeliz Prior) have experience with conducting qualitative research in musculoskeletal pain and arthritis.

## 3 RESULTS

Forty‐three potential participants were approached, 26 declined or did not respond to the interview study information, one participant was ineligible at screening and one participant was eligible, but did not attend their interview and did not respond after being re‐contacted.

Fifteen participants were interviewed (Mean age: 56.8 years old [range 40–65]; Female [60%]) (Table 2). Twelve participants reported having OA in multiple joints, with the knee joint being the most common and then the hip and hand joints. Two participants reported having joint replacement surgery due to OA. The one‐to‐one telephone interviews were on average 47 min in duration (range of 23–78 min). No repeat interviews were carried out.

**TABLE 2:** Participants' demographics.

Participant ID and pseudonym	Sex	Age, years	Joint pain on activity?	Multi‐joint OA?	OA joint(s) and year of diagnosis	Joint replacement surgery due to OA?	Joint replacement joint and year	Job title	ONS job skill level
01 Jane	F	50	Y	Y	L knee 2012; L & R hip 2010; L & R hand 2012	Y	R hip 2011	Accountant	4
02 John	M	57	Y	Y	L & R hand 2016	N	N/A	Production manager—mechanical engineer	4
03 Danny	M	40	Y	Y	L & R knee 2018	N	N/A	Car manufacturer associate—builds cars	3
04 Milo	M	62	Y	N	R knee (do not remember)	N	N/A	Account manager in warehouse	3
05 Janet	F	61	Y	Y	L & R knee 2009	N	N/A	Senior healthcare assistant	2
06 Boris	M	45	Y	Y	L & R hip 2018	N	N/A	Watch manager—fire station at the airport	3
07 Claudia	F	59	Y	Y	L & R knee 2017; L & R hip 2018; L & R hand 2005	N	N/A	Scientist	4
08 Maggie	F	55	Y	Y	L & R knee 2018; L & R hip 2018; L & R hand 2017	N	N/A	Laundry assistant	1
09 Jacqueline	F	64	Y	Y	L & R knee 2017; L & R hip 2000; L & R hand 2018; neck 1990	N	N/A	Postal manager in distribution centre	4
10 Jay Jay	F	54	Y	Y	L knee 2013; R knee 2017; L hip 1993; R hip 2002; L & R thumb 2017	Y	L hip 2001; R hip 2009; L knee 2018; R knee 2014	Healthcare administrator	2
11 Anna	F	65	Y	N	R hip 2019	N	N/A	University administrator	2
12 Monica	F	58	Y	Y	L & R hand 1999; L & R foot (toes) 2015	N	N/A	Newborn hearing screener NHS	3
13 Audrey	F	60	N	Y	L & R knee 2009; L & R hip (don't remember); lower back (don't remember)	N	N/A	Learning and disabilities support worker	2
14 Kenneth	M	58	Y	Y	L & R hip (don't remember); lower back 2014	N	N/A	Civil servant (driver)	2
15 Jack	M	64	Y	Y	L & R knee 2009; L & R wrist 2019	N	N/A	Warehouse officer	1

*Note*: Office for National Statistics job skill: Level 1 (elementary occupations); 2 (Administrative, caring, leisure, sales, customer service; process, plant and machinery operatives); 3 (Associated professional and technical/skilled trades); 4 (Professional and managerial) (website: accessed: 11 November 2022 https://www.ons.gov.uk/methodology/classificationsandstandards/standardoccupationalclassificationsoc/soc2010).

Abbreviations: F, female; ID, identification number; L, left; M, male; N, no; N/A, not applicable; NHS, National Health Service; OA, osteoarthritis; ONS, Office of National Statistics; R, right; Y, yes.

### 3.1 Effects of OA on work participation (Table 3)

**FIGURE 1:**
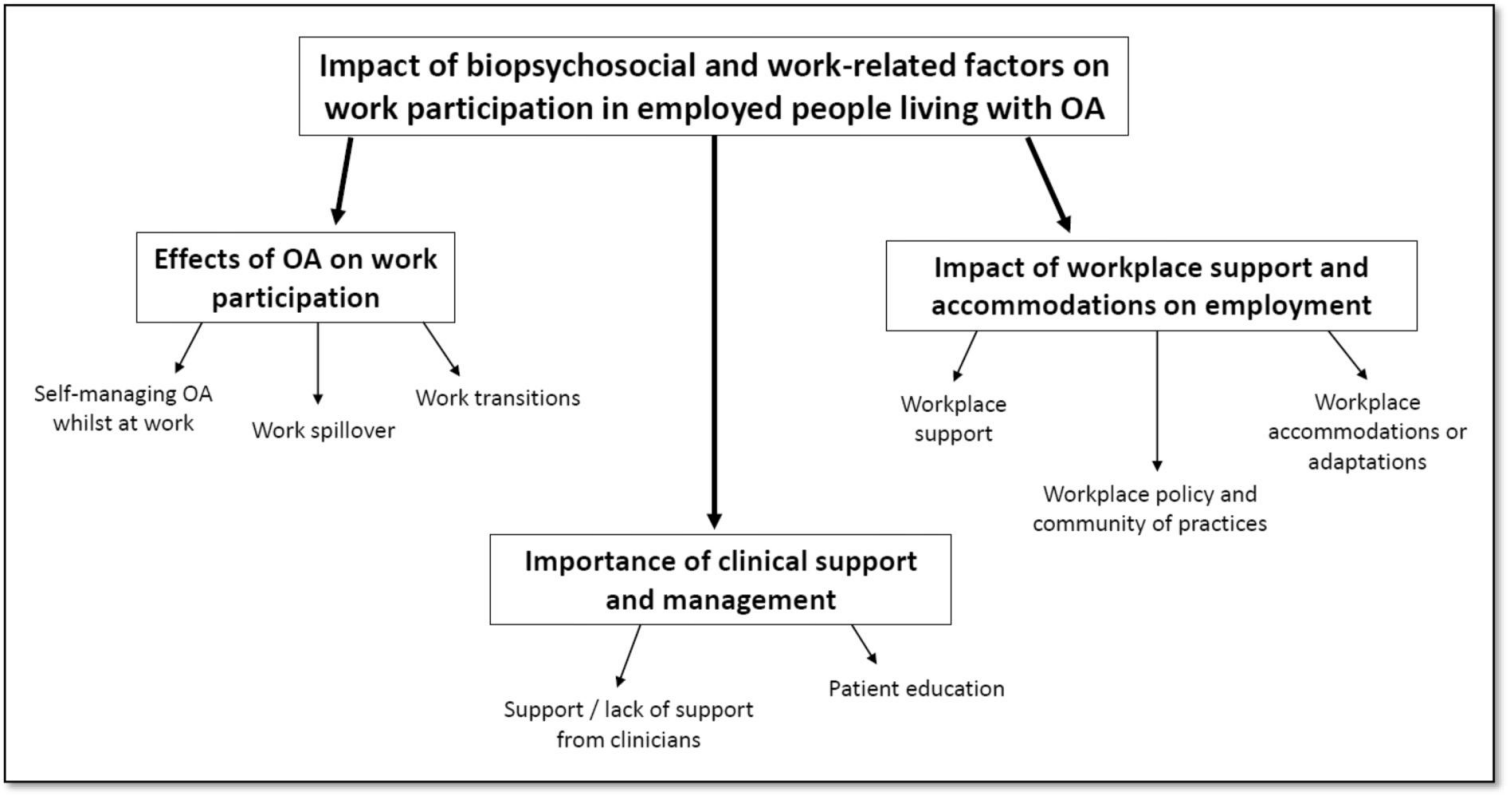
Themes coding tree.

Participants described how OA impacted on their work and how they self‐manage their condition whilst at work. Some participants described the work transitions (i.e., work interruptions), such as being unable to take on extra responsibilities; lost time at work (due to leaving work early, arriving late or taking an extended lunch break or extra breaks) (Gignac et al., 2008). Participants also reflected on the impact of work spillover, which is defined as the stresses experienced in one area of life (e.g., work and/or home life) lead to stresses in another area of life (e.g., managing OA), and vice versa (Gignac et al., 2006) (Figure 1 shows the coding tree).

**TABLE 3:** Quotes from Theme 1 and its sub‐themes.

Theme 1: Effects of OA on work participation	
Self‐managing OA whilst at work	
Claudia, 59, Scientist	*‘I'm going to be 60 this year…but I think next year I probably just won't be able to do harvest and I would have had to get somebody else to do it, because I physically won't be able to do it, which is a horrible admission of defeat, that's part of being admission of defeat and that's why people perhaps push themselves for longer than they should?’*
Kenneth, 58, Civil Servant	*‘I suffer from migraines at a level where, if I get a migraine, I wouldn't even know what my children's names are, and I'm physically sick with that’.*
Jacqueline, 64, Postal Manager	*‘I have pain all the time, I just switch off to it. The only time that I find it bothers me is when I'm in bed trying to get to sleep… But during the day, I'm working with pain, but I just, because you're occupied…you just push it away and you carry on’.*
Work transitions	
Jane, 50, Accountant	*‘I couldn't hands on treat, I couldn't have done my Occ Health job 5 days a week…I could have tried to go into management, you know, but I couldn't have done the classic MSK physio [musculoskeletal physiotherapy] job’.*
Jane, 50, Accountant	*‘The flexibility [of the new career] means I don't stress about it. It's kind of stressful enough when you have to say, “I'm really sorry, I can't work today”, without all the sick notes and reviews going on yeah that come with that’.*
Jay Jay, 54, Healthcare Administrator	*‘When I started the job, it was full‐time and I did ask after a few months about going part‐time, but they said “no, it wasn't possible”, due to the fact that if I cut my hours, they would lose the time’.*
Audrey, 60, Learning and Disabilities Support Worker	*‘Working with learning disabilities, that's all I know to be honest…like, me job role has always been round [learning disabilities]’.*
Work spillover	
Jay Jay, 54, Healthcare Administrator	*‘Yeah, I've had to cut my hours down, I was finding it too much just working full‐time. I just found it compacted on the rest of my week. I spend the weekend just feeling I've absolutely had it, and really dreading going back the whole week’.*
Jane, 50, Accountant	*‘The harder jobs with the cleaning were having a negative impact in that they increase pain, inflammation, and fatigue, and therefore using my energy or my joints for cleaning was not letting me then use it for other things’.*
Maggie, 55, Laundry Assistant	*‘I tend to pace myself, I would come home, and I would do some housework practically every day, so one day it might just be hoovering, the other day it might be dusting’.*

*Note*: The italics is to emphasise direct/ verbatim quotes from the participants.

#### 3.1.1 Self‐managing OA whilst at work

Joint pain was the most common symptom reported, with 13 participants living with pain in multiple joints. Participants found it more problematic to manage their joint pain if the work tasks required them to use their painful joint(s). This also caused some concerns in their work performance, as they worried that they may not be fit enough to do the physical tasks required for their job anymore.

Some participants also lived with comorbidities such as migraines, cancer, menopause, or high blood pressure and they felt that these also affected their work productivity. Sometimes, managing their other conditions was more stressful compared to managing their OA. For example, Kenneth felt that his migraine pain can be more painful and severe than his OA pain.

Participants understood their OA symptoms and how this affected their work and home lives, they knew their limits and used active or passive coping strategies to help them stay in employment. For example, some participants were able to delegate physically demanding tasks that caused strain on their joints to their colleagues, used pain killers, had acupuncture, cortisone injections. Most participants had tried exercise, being physically active and/or attended exercise programmes led by physiotherapists through the National Health Service (NHS) or privately. Whilst other participants found it easier to ignore the pain and carry on.

#### 3.1.2 Work transitions

Participants described making different work transitions, such as changed job/employer, changed work hours or changed job roles with the same employer. Jane and Kenneth discussed re‐training from careers that were physically demanding to less physically demanding careers. This change was positive in enabling them to remain in employment. For instance, Jane previously worked as a physiotherapist but decided to re‐train as an accountant. She was aware that her hand pain and OA would not allow her to keep working as a full‐time physiotherapist until retirement. So, she chose a new career as an accountant which required less physical demand on her hands and allowed her more control and flexibility in her work schedule.

Half of the participants reduced their work hours due to their ‘condition getting worse’ or ‘tiredness’ to allow for more time for rest and relaxation. However, this option was not always possible for all participants, as reduction in hours also meant a reduction in their salary or their workplace did not allow this.

Monica and Audrey felt that they were restricted to the jobs they could do, as they both had specialised jobs (i.e., a new‐born hearing screener and learning and disabilities support worker, respectively).

#### 3.1.3 Work spillover

Stresses experienced from participants' home/work life also led to stresses in another area of life, such as managing their OA and vice versa. Participants who experienced work spillover, also made adaptations in their home life to help manage their OA symptoms. For example, some participants who worked part‐time, felt having rest days between workdays was important in allowing them to recover.

Doing household chores was difficult for some, as it increased their joint pain, inflammation and caused fatigue. Participants reflected that they were often too tired to complete their chores at the end of the working week.

Participants had different strategies for household chores. Monica and Jane could afford to pay for a cleaner to help with household chores, as this allowed them more time to rest and recover. Others reflected on the importance of pacing and splitting up their chores, so they had smaller chores to do each evening rather than saving it all for the days off.

### 3.2 Impact of workplace support and accommodations on employment (Table 4)

In this theme, participants described the support or lack of support from their employers and colleagues, their awareness of workplace policies and their rights at work and the workplace culture. Participants also reflected on the availability (or lack of) of making workplace adaptations and/or accommodations (Figure 1).

**TABLE 4:** Quotes from Theme 2 and its sub‐themes.

Theme 2: Impact of workplace support and accommodations on employment	
Workplace support	
Jack, 64, Warehouse Officer	*‘I had one manager…she was brilliant, I could talk to her, I could tell her when I needed to go to the clinic, she'd give me time off, and she was very, very supportive. But I'm afraid she's moved on to another section now. And at the moment, we have a lady in charge, who is not as sympathetic, shall I say’.*
Workplace policy and community of practices	
Jane, 50, Accountant—this quote was reflecting on time as a physiotherapist	*‘I didn't take any sick leave because of it [OA]. Did it mean that some days I did very little? Yes, absolutely. I didn't take any arthritis‐related time off. I'm very scared of it’.*
Janet, 61, Senior Healthcare Assistant	*‘I have [taken sick leave]. And it's got me into lots of trouble’.*
Maggie, 55, Laundry Assistant	*‘I personally don't think anyone would employ me…I think that as soon as you put on your application form that you've got health issues…I don't think employers would want to know…I think that they would have concerns about whether you could do the job, the time off that you might take’.*
Jacqueline, 64, Postal Manager	*‘I don't want to show that I can't do the job, so you feel like, part of the fact that I'm the oldest, part I'm a woman, so you [are] constantly trying to prove yourself’.*
Claudia, 59, Scientist	*‘I did think, part of the problem is me, or people like me who still keep pushing when we probably should stop or do less…I can't blame work for everything, I mean, it is partly an attitude, a way of working that comes from me and the other people of my kind of vintage and mind set’.*
Maggie, 55, Laundry Assistant	*‘Trouble is, like a lot of people, if I don't go into work, I don't get paid’.*
Workplace accommodations or adaptations	
Jack, 64, Warehouse Officer	*‘I think you try and make your job simple and easy, like…if we find a problem, instead of taking that right away and having to walk a long, long way, we'll wait until we have several problems before we go. I know it sounds pretty basic, but you're only making one trip rather than making several trips’.*
Jay Jay, 54, Healthcare Administrator	*‘They were really good. I had been off work for a long time…I'd had an operation that had gone wrong, and it had taken me a long time to get back into work. Access to Work had got involved with helping bring me back into the workplace, and I found they were very, very helpful’.*
John, 57, Production Manager—Mechanical Engineer	*‘When I'm using measuring equipment to measure parts, you have to use your thumb and forefinger quite a lot for checking parts. If I have to do that probably for about an hour, I find that…at the end of the day it's quite achy, so rather than doing that all in one go, I'll probably spread that…so I don't get that pain’.*
Maggie, 55, Laundry Assistant	*‘To be honest because the job is what it is. I don't really see what they could do to help me with my job to be honest. It's not as though they could move the laundry closer to the door or anything like that’.*

*Note*: The italics is to emphasise direct/ verbatim quotes from the participants.

#### 3.2.1 Workplace support

Having a good employee–manager rapport played an important role as to whether participants felt comfortable disclosing their condition to their manager or employer. Some participants disclosed their condition to their employer and had open discussions about the tasks they could not perform due to their OA. Whilst others only felt comfortable telling their employers the minimum information about their condition.

Jack found it more difficult to connect with his current line manager and was not comfortable with disclosing his condition to them. He compared his current manager with his previous manager, who was more sympathetic, approachable and supportive.

Participants who had good working relationships with their colleagues felt supported and understood by them, as they were flexible and offered to help with tasks that participants found more challenging due to OA.

#### 3.2.2 Workplace policy and community of practices

Half of the participants interviewed spoke about taking sick leave due to their OA, whilst the other half said that they did not need to take time off due to their OA. For those that did take time off, some felt unable to take more sick leave, because they feared the consequences that could occur, as they had already taken too much sick leave.

Going through the sick leave process at work was stressful for some participants, as they were aware that this will go on their record and if they exceed the sick leave limit then this could lead to a review with Human Resources.

Participants reflected on not wanting to change from their current job/employment because they felt they would be discriminated against if they disclosed their long‐term health condition, as they may be seen as an ‘unreliable employee’.

A couple of participants in managerial positions felt they had something to prove to their subordinates, because they did not want to be seen as incapable at their job.

People went to work even when they knew they may not be 100% productive for different reasons. For a lot of participants, it was their work ethic to keep working and not take time off for sick leave. It could also be culture of their workplace where they needed to work longer hours than contracted, which puts a strain on the body and mind.

Even where participants were unwell, they reflected on turning up for work because of their work ethic and they did not want to let others down. However, participants reflected that this could lead to colleagues and employer wrongly assuming the participant was fine, because they had turned up for work.

Furthermore, some participants were breadwinners and had dependants, so needed to keep working.

#### 3.2.3 Workplace accommodations or adaptations

Participants discussed using different workplace accommodations or adaptations that enabled them to stay in employment. Many participants' work tasks could be adapted by changing the way they performed tasks or buying ergonomic equipment or workwear to help manage their symptoms. For example, Jack worked in a large warehouse and his work involved a lot of walking and collecting/packing items for customers. He used a trolley to help lighten the weight when carrying stock around the warehouse.

Jay Jay had support from Access to Work, which allowed her return to work to be smoother. Her workplace had arranged for an ergonomic chair and phased return to work.

Half of the participants were in job roles that were flexible or varied, which helped with their productive and gave them more control, as they could pace themselves and split their tasks throughout the day.

Other participants had job tasks which were less flexible. For example, Maggie worked as a laundry assistant in a care home and the nature of the job, and its tasks meant that finding alternative ways of performing certain tasks were not possible. Additionally, she was also the only person working on her shift, so did not have support of colleagues to help with tasks.

### 3.3 Importance of clinical support and management (Table 5)

Clinical support received impacted on participants' understanding of their condition and how to effectively manage their symptoms. This theme explored how supported participants felt from their clinicians and patient education around the management of OA (Figure 1).

**TABLE 5:** Quotes from Theme 3 and its sub‐themes.

Theme 3: Importance of clinical support and management	
Support/lack of support from clinicians	
Boris, 45, Fire Station Watch Manager	*‘I know the NHS GPs are under a massive pressure and they've got their own targets and stuff, but I just kinda felt that…once he'd pushed me away for the x‐ray, that that was him having dealt with it. I don't feel that the aftercare was very good’.*
	When Boris was asked about what he would have wanted in terms of support, he said:
	*‘I guess I would have liked to see a bit more, “why don't you try this going forward”, or “have you thought about trying this”, or “there is this as an alternative”, do you know what I mean?’*
Anna, 65, University Administrator	Similarly, Anna also felt a lack of time and support from her healthcare professionals.
	*‘I was very, very disappointed with the GP when I first went…she said she only had 5 min and gave me some literature to read…I was very, very disappointed and waiting 6 months for a physio appointment, again, they did an assessment and said “yeah, ok, off you go, here are your exercises” and that was it’.*
Anna, 65, University Administrator	*‘I did find one wonderful thing, a 12‐week joint pain programme…£2 a session, you go and use the gym and you do exercises and that was quite good because I met other people with similar things in the hips or bad knees, so that was really quite good. And again, I stumbled on to it without anybody saying anything’.*
Patient education	
Anna, 65, University Administrator	*‘I really, really appreciated just a little bit more information, any “dos and don'ts’.*
Maggie, 55, Laundry Assistant	*‘Yes, they provided leaflets, etc. There's so much information concerning how you can help yourself if you've got arthritis, even down to the diet, but some of it does contradict itself a bit, I think it's picking the things that you want to try, and which might be beneficial for each person’.*
Jack, 64, Warehouse Officer	*‘Yes, I think it has been very good, because the last time I saw a lady who was helping me with my hands, giving me the supports…she was very, very good. I learnt more from her than I had done from my doctor over years with my knees’.*

*Note*: The italics is to emphasise direct/ verbatim quotes from the participants.

#### 3.3.1 Support/lack of support from clinicians

A couple of participants felt their general practitioners (GPs) were supportive towards the management of their OA. However, a few participants felt a lack of support from their clinicians, lack of follow‐up and a dismissive attitude about the management of OA and treatment advice. When Boris was asked about what he would have wanted in terms of support, he suggested having more meaningful follow‐up conversations with health professionals on the progression of his condition.

Similarly, Anna also felt a lack of time and support from her healthcare professionals. After her disappointment, Anna found a 12‐week joint pain programme through a local health centre, which she paid for and attended. The support she received from the instructor and peers in the programme demonstrates the importance of how different attitudes of professionals to treatment and management of OA can influence a person.

#### 3.3.2 Patient education

Some participants felt that the information for self‐management of OA for patients was unclear and access to good quality information was lacking.

However, Jack and Boris reflected they were pleased they received support from physiotherapists who explained their condition to them, why they were experiencing pain and what they could do to reduce their symptoms. Having more positive and supportive relationship with healthcare professionals meant these participants had a better understanding of their symptoms and self‐management strategies.

## 4 DISCUSSION

### 4.1 Effects of OA on work participation

OA had physical and mental impact on people's health and work productivity. Participants from our study were proactive in their efforts of remaining in employment through using different coping strategies. A mixed‐methods study by Oakman et al., explored work and non‐work support used by employees living with persistent musculoskeletal pain to assist them in maintaining productive employment (Oakman et al., 2017). The authors found that despite pain and disability, presenteeism was moderate and limited productivity loss, suggesting that coping strategies and workplace accommodations used helped keep participants in productive employment (Oakman et al., 2017). Similarly, our study also showed that participants who found their jobs physically challenging were proactively using different coping strategies and adaptations such as, using different equipment and aids, ergonomic equipment and modifying work tasks to ensure they could carry out their tasks.

Various work transitions may occur in the work lives of people living with arthritis, with factors such as age, sex, education, depression, activity limitations, control and arthritis‐work spillover being associated with work transitions (Gignac et al., 2008). Some participants in our study reflected on making work transitions (i.e., absenteeism, reduce hours or changed jobs), whilst others were unable to due to the nature of their job. Likewise, a previous study found that people with OA or Inflammatory Arthritis (IA) in occupations with specialised training and the nature of these jobs may make work transitions (i.e., reduced hours and changing jobs) less feasible (Gignac et al., 2008). This highlights the importance of understanding if and how employed people living with OA make work transitions and the impact or barriers these work transitions has had on maintaining employment, as previous research indicate that more work transitions were made by people with arthritis who perceived their condition had a significant impact on their work abilities (Gignac et al., 2004). However, we know that not everyone is able to make work transitions.

Participants who experienced work spillover made adaptations in their home lives to allow more time to rest and recover, which helped with their productivity at work. In addition, people with knee OA balanced the risks and benefits of physical activity and exercise, such as balancing ‘safe’ activities with activities they enjoyed while also planning their lives around their condition (Darlow et al., 2018).

### 4.2 Impact of workplace support and accommodations

Our study found that some people disclosed their condition to their employer/line manager, and some did not. Participants were less comfortable with disclosing their condition with their manager if they did not have good rapport with them or if the manager seemed dismissive. This finding was similar to another recent qualitative study that explored work‐related experiences of younger people living with IA or OA (Berkovic et al., 2020). Berkovic et al., found participants were hesitant to disclose their condition to their employer or co‐workers due to the fear of unequal treatment in the workplace. However, another study found that those who discussed their arthritis with their employer or colleagues found that they were understanding and supportive (Berkovic et al., 2020). Interestingly, research from Gignac et al., described that people who perceived it was safe to disclose their condition to their employer (i.e., they would remain employed) reported more work changes when they disclosed their condition than when they had not (Gignac et al., 2004). Having the knowledge of available workplace support to assist staying in employment can help employed people living with OA weight up the risks and benefits of disclosure of their condition to their employer (Oakman et al., 2017).

These findings complement previous studies in fibromyalgia and chronic illness that found healthy workplace relationships and positive workplace culture increased an individual's self‐efficacy to reveal their condition and to discuss required support and workplace accommodations (Lindsay et al., 2018; Munir et al., 2005; Oldfield et al., 2016). Thus, more should be done through educating employers and employees to improve workplace relationships and increase individual's self‐efficacy to disclose their conditions and receive support and workplace accommodations.

Participants in our study used workplace accommodations such as reduced or changed working hours/shifts; ergonomic workspace set up; or modified job duties. Those who had flexible work schedules felt more in control at managing their work and their condition. Furthermore, Gignac et al., found that less control over work schedule was associated with more arthritis‐work spillover or leaving employment in individuals with IA or OA (Gignac et al., 2006, 2008).

Some participants in our study experienced the pressure of staying in the same job, because they felt they would be discriminated against if they disclose their long‐term health condition, as they may be seen as an unreliable employee. Additionally, those in jobs with specialised skills felt less able to change careers. Similarly, previous research have found that people with IA or OA who were healthcare professionals (e.g., nurses) or teachers with specialised training and the nature of their jobs may make it more challenging to have work transitions such as reduced hours or changing jobs (Gignac et al., 2008).

### 4.3 Importance of clinical support and management

Our study found the differences in health professionals' attitudes to treatment and management of OA can impact individuals' attitude and knowledge about the management of their condition at work, and in their daily lives. A positive and supportive approach could lead to increased productivity at work. Our findings build on work from previous studies, which found that health professionals may trivialise OA and often associated it with ‘wear and tear’ and as part of ageing (Campbell et al., 2001; Sanders et al., 2004). This in turn could lead to people with OA becoming pessimistic about engaging with health professionals about their condition or receiving inaccurate information (Darlow et al., 2018). Thus, there is a need to address the inaccurate language used and beliefs around the management of OA (Darlow et al., 2018).

Even though participants in our study reported that information for self‐management of OA was unclear and access to good quality information was lacking, participants utilised a range of coping strategies and work adaptations to carry out their work tasks and maintain employment. In the United Kingdom, there are reliable resources and work‐related support that people with long‐term health conditions can receive. For example, Access to Work can provide a grant to help pay for practical and mental health support at work (GOV.UK, 2022); the Equality Act 2010, which provides legal protection for people from discrimination in the workplace and in wider society (GOV.UK, 2010); and the National Institute for Health and Care Excellence (NICE) has published Quality Standard guidance for employers on how they can support the health and wellbeing of their employees with long‐term health condition, who are taking or retuning from sickness absence (National Institute for Health and Care Excellence, 2021). It is important that employees with OA, colleagues and employers are aware of these resources and educational materials available to support people in the workplace who live with OA. This will give individuals with OA more control and flexibility with their work arrangements and access workplace accommodations to support productivity at work and remain in employment (Oakman et al., 2017).

### 4.4 Strengths and limitations

There are strengths and limitations in our study. A strength was our sample included people with OA in different joints and people with different careers, which allowed for us to explore the range of perspectives of how OA impacts their work lives. Using telephone interview method also gave us access to people living across the United Kingdom. Findings from our study added new perspectives on work transitions (made or considered), the effects of work spillover into other areas of people's lives, and the impact of workplace accommodations on employed people living with OA.

A limitation in our study was the use of convenience sampling rather than purposive. Although this method of sampling was low‐cost, quick and easy, as participants were readily available from a volunteer database, it did limit the pool of people we approached. The social media recruitment approach used to counteract this was unsuccessful with very low numbers of potential participants expressing interest in the study. Therefore, there may be an under‐representation of perspectives from those who were not approached about the study, that is, our sample only included two people in job skill level 1 (elementary jobs).

## 5 CONCLUSIONS

Our findings illustrate that OA impacts on people's work productivity and work spillover into their home lives. Participants have used workplace accommodations, made work adaptations, and work transitions to remain in employment. However, these work changes are not always possible due to the nature of people's jobs. This highlights the importance of understanding the nature of employment and having knowledge of available workplace support to assist people to remain in employment.

In the United Kingdom, reliable resources and work‐related support are available for people with long‐term health conditions. However, participants in our study reflected that access to good quality resources and information is lacking. Thus, more attention is needed on finding an effective way to distribute reliable information and resources on work‐related support to employed individuals with OA and their employer. By creating more awareness and education around working with OA, this could give individuals with OA more control and flexibility with their work arrangements and access workplace accommodations to support productive work and maintain employment.

## AUTHOR CONTRIBUTIONS

Yeliz Prior conceived the study. Both authors have made substantial contributions to the design. Angela Ching led the acquisition of data and analysis. Both authors contributed to the interpretation of the data and writing the manuscript.

## CONFLICT OF INTEREST STATEMENT

All authors declare they have no conflicting interests.

## ETHICS STATEMENT

The University of Salford Health Sciences Research Ethics Panel approved the study (HSR1819‐008) and written informed consent was received from all participants.

## Data Availability

The data that support the findings of this study are available from the corresponding author upon reasonable request.
